# Exploration of inter-jurisdictional TB programming and mobility in a Canadian First Nation community

**DOI:** 10.1186/s12889-022-14756-8

**Published:** 2022-12-14

**Authors:** Apeksha Heendeniya Vidanaral, Richard Long, Courtney Heffernan, Sylvia Abonyi, Sherry Clarke, Paul Hackett

**Affiliations:** 1 University of Saskatchewan, Kirk Hall Building, 117 Science Place, Saskatoon, SK S7N 5C8 Canada; 2 Department of Medicine, University of Alberta, 8333A ABC Aberhart Centre, 11402 University Avenue, Edmonton, AB T6G 2J3 Canada; 3 University of Alberta, 8326 ABC Aberhart Centre, 11402 University Avenue, Edmonton, AB T6G 2J3 Canada; 4 Health Sciences Building, University of Saskatchewan, 104 Clinic Place, Saskatoon, SK S7N 2Z4 Canada; 5 La Loche Community Health Centre, Dene Rd, La Loche, SK S0M 1G0 Canada

**Keywords:** Tuberculosis, First Nations, Health Geography, Mobility Studies, Policy Analysis

## Abstract

**Background:**

Colonially imposed jurisdictional boundaries that have little meaning to Indigenous peoples in Canada may confound tuberculosis (TB) prevention and care activities. This study explores how inter-jurisdictional mobility and the current accommodation of mobility through policies and programming sustain a regional TB epidemic in northwestern Saskatchewan, and northeastern Alberta.

**Methods:**

A qualitative instrumental case study was performed using a community based participatory approach. Semi-structured interviews were conducted with First Nations peoples from a high-incidence community in Canada including community-based healthcare workers. These interview data are presented in the context of a multi-level document analysis of TB program guidelines.

**Results:**

The location of the community, and related lack of access to employment, services and care, necessitates mobility across jurisdictional boundaries. There are currently no formal federal or provincial guidelines in place to accommodate highly mobile patients and clients within and across provincial TB prevention and care programs. As a result, locally developed community-based protocols, and related ad-hoc strategies ensure continuity of care.

**Conclusion:**

Indigenous peoples living in remote communities face unique push/pull factors that motivate mobility. When these motivations exist in communities with increased risk of contagion by communicable infectious diseases such as TB, public health risks extend into increasingly large areas with competing jurisdictional authority. Such mobility poses several threats to TB elimination. We have identified a gap in TB services to systematically accommodate mobility, with specific implications for Indigenous peoples and reconciliation. We recommend clearly defined communication paths and inter-jurisdictional coordination to ensure maintenance of care for mobile populations.

**Supplementary Information:**

The online version contains supplementary material available at 10.1186/s12889-022-14756-8.

## Introduction

Tuberculosis (TB) is a communicable infectious disease transmissible by the aerosol route. With the temporary exception of COVID-19 (the disease caused by SARS-CoV-2) in 2019–21, TB has been the number one infectious disease killer globally [1]. Despite this distinction, the rate of disease in high-income countries remains relatively low. Canada is no exception, where the annual rate of disease has been steady around 5 per 100,000 population for the last two decades [2]. The burden of disease, however, is very unequal. Foreign-born persons from high-incidence countries report the highest number of total TB patients annually; and, Indigenous peoples, a subpopulation comprised of three distinct groups – First Nations, Métis, and Inuit – experience the highest annual rate of disease [3]. In 2017, the incidence of TB among Canadian-born First Nations was 34.2 times that of Canadian-born non-Indigenous persons [4]. For Indigenous peoples of Canada, the roots of this unequal burden of disease stem from the past.

The impact of colonialism, which established and continues to maintain overcrowded and congregate living in small communities, lack of economic opportunity, and lack of healthcare access is contributory to this unequal burden of disease [5–7]. These conditions have led to both excess exposures and increased risks for reactivation that sustain TB in many Indigenous communities. In addition to a disparate experience of disease by population, there are unequal notifications by geography. Across the Canadian prairies, individual community experiences with the disease range from near or total absence punctuated by periodic outbreaks, to endemicity [8]. In the Prairie Province of Saskatchewan, 54% of the incident TB patients are First Nations, and most live in the far north zone [9]. Mobility in and out of communities is likely to occur where access to essential services is scarce. The coupling of mobility to infectious disease has the potential to worsen patient outcomes, thereby exacerbating the health gap between Indigenous and non-Indigenous Canadians and to confound TB elimination efforts overall. In fact, frequent travel has been identified as an obstacle to obtaining health care and factored into interruption in TB therapy but is an understudied phenomenon [10, 11]. The purpose of this paper is to explore the possible role of TB programming differences and inter-jurisdictional mobility in sustaining the high incidence of TB in Indigenous communities. We use qualitative interview data and document analysis with a focus on a cluster of communities in Saskatchewan and the neighbouring Province of Alberta. We discuss the implications of our findings for the effective delivery of TB prevention and care services considering these data.

## Background

On the Canadian prairies today, TB impacts specific communities with high incidence, where one or more determinants such as malnutrition, food insecurity, poor housing, overcrowding, environmental conditions, and access to resources and healthcare, may be having an impact. These determinants can influence TB pathogenesis by increasing exposure to active TB and the likelihood of infection progressing to disease. Insufficient access to resources and healthcare can lead to lack of diagnosis and poor treatment success [12]. Contemporary TB patient experiences among First Nations have common themes, such as a struggle accessing basic needs, stigma surrounding a TB diagnosis, and social isolation [13–15]. One factor that has received little attention, but which nonetheless may play a role in the inequitable occurrence of TB among Indigenous people in Canada, is the movement of people through space, an element of human activity that is understood by geographers as a fundamental component of human health.

Mobility and migration, although often used interchangeably, are different concepts. Both refer to movement across space, with migration involving a long-term, often permanent, change in residence or community [16]. Short term mobility is defined as movement out of the home community for one week or less, while long-term mobility is an absence for a period longer than one week and up to one month. Motivations for both migration and mobility are complex and reflect forces operating at multiple levels. Some, especially short-term, Indigenous mobility factors relate to work, education, traditional ceremonies, and returning to family [17, 18]. Others, such as increased urbanization or kinship networks connecting different communities, could be considered a continuation or extension of traditional mobility patterns [19–21].

Historically, Indigenous peoples living in what is now Western Canada, were seasonally mobile within their lands as they pursued traditional economic activities (e.g. hunting and trapping) but also as they moved to key intersectional locations where larger groups formed for social, political, and resource reasons [22]. Alienation from the land and these vital movements has for some contributed to ongoing poor health [23]. Jurisdictional borders that divide reserves from surrounding communities and provinces from one another often cross traditional Indigenous routes of travel, and kin networks, and are thus continually traversed. First Nations people living on-reserve are under a federal system of healthcare. But the reserves are located within provinces and territories, each of which has its own healthcare delivery system – and with respect to TB, its own TB program. There is no national program per se. When it comes to TB services on-reserve, the Federal government has worked out provincial/territorial – unique service delivery arrangements. In the Province of Saskatchewan, they support or contract with the Northern Intertribal Health Authority (NITHA), a transferred organization, to deliver TB services on-reserve. Service delivery in other communities is solely under the jurisdiction of the provincial government.

There may be unintended consequences to a high degree of mobility across a territory divided into multiple jurisdictions. For example, incursion of infectious agents like *Mycobacterium tuberculosis* (the organism that causes TB disease) across communities with highly mobile residents is cause for concern [24].

In a 2010 study of inter-jurisdictional transmission of TB among Canadian-born Indigenous and non-Indigenous individuals, Aspler et al. (2010) found the same strain of *M. tuberculosis* implicated in cases over an area of 26,000km^2^. Transmission was believed to have been facilitated by the high degree of mobility among the cohort, ever more likely in the presence of a delayed diagnosis, which despite the presence of symptoms, frequently occurs among Indigenous TB patients on the Canadian Prairies [14, 25].

The present study sought to explore how interjurisdictional mobility and the current accommodation of mobility through policies and programming may sustain a regional TB epidemic in Northwestern Saskatchewan and Northeastern Alberta, Canada.

## Methods

We carried out a qualitative instrumental case study which began in 2018 with completion in 2020 within a much larger Canadian Institutes for Health Research (CIHR) funded implementation science study titled, *Implementing the “Patient’s Charter of Tuberculosis Care” in high incidence Indigenous communities and across jurisdictional borders* (hereafter “Pathways”) [26]*.* Pathways has heretofore been undertaken in four high incidence communities located near the provincial border between Alberta and Saskatchewan. In all its components it spans the years 2015- 2024. Community partnership and collaboration as per the principles of Community-based participatory research (CBPR) are integral to this study. Through the Pathways project a TB research committee of community stakeholders, government personnel and academics was formed to provide guidance for the project and research objectives. This was further enhanced with two subcommittees, one representing the two communities in Alberta and another representing the two communities in Saskatchewan. Community stakeholders include persons from two Dene First Nations, many of whom, in addition to being residents, work as health care providers. Community-research committees include other advisors, such as Elders, knowledge-keepers, youth, and current/past TB survivors.

The Saskatchewan subcommittee raised concerns about inter-jurisdictional mobility and a need to understand the functional reality of TB care in border communities. The committee provided feedback on the study design and was contacted throughout for advice about iterations to methods and results. The chair of this committee, who is a collaborator on Pathways is a co-author of this study article. Ethics approval was granted by the University of Saskatchewan’s Research Ethics Board ID 908.

The specific tools for gathering data included semi-structured interviews, and an instrumental case study approach. Semi structured interviews (Additional file 1: Appendix 2) were undertaken by a graduate student researcher. They allowed First Nations community participants to share their stories openly, reflecting Indigenous storytelling research methods [27]. Storytelling is an important practice in Indigenous cultures and provides opportunities to express experiences of Indigenous peoples. Indigenous ideologies are connected to community and land, community members chose the most comfortable location of interviews and often chose their homes as locations. Indigenous epistemology is grounded in the interconnectedness of the physical, mental, emotional, and spiritual [28]. With regards to positionality, the researcher lived in the community for weeks at a time, participating in community events, gatherings, and traditional practices to develop a trusting relationship with the community, while learning from individual members. At the same time, the researcher reflected on her own positionality by maintaining a journal throughout the study.

Interviewees included those who self-identified as First Nations from a reserve community (hereafter, “R”) near the border between Alberta and Saskatchewan. Other interviewees included participants from a neighbouring northern village (hereafter, “C”) to contextualize the close relationship between R and C. R is located 7 km from C, and about 37 km from the provincial border (Fig. 2). In 2016, community R and C had populations of 800 + and 2000 + , respectively. For persons aged 15 and older and living in private households, the median income was $15,328 in community R, $21,043 in community C, and $38,299 in the Province of Saskatchewan overall. Education was limited with only one-third of the persons 15 years of age or older having a high school diploma, certificate, or degree. The unemployment rate was 44.4% in community R and 27.6% in community C versus 7.1% in the province overall. In community R, the focus of the study, the number of private households that had more than one person per room was 18.4% versus 1.9% in Canada overall. According to the National Occupancy Standard (NOS), 44.7% of the private households (versus 4.9% in Canada overall) in the community were considered unsuitable; that is the dwelling did not have enough bedrooms for the size and composition of the household [29]. Across communities R and C, the incidence of TB was 148 per 100,000 persons in 2019. Amongst TB contacts that are screened in Community R and C and determined to have active TB or latent TB infection, treatment outcomes are known to be suboptimal.

Because TB is stigmatizing and, for Indigenous peoples in Canada, fraught with historical trauma [5, 15, 30], inclusion criteria for interviewees was not limited to current or recent-past TB patients, but more broadly invited stories about mobility from anyone in the community aged > 17 years of age. Data was collected in two phases that corresponded to our dual objectives. First, we conducted interviews related to motivations for and patterns of mobility. Second, we examined the response by TB services to the phenomenon with a document analysis of national and provincial guidelines, and by conducting interviews with healthcare workers for further detail. As above, several healthcare providers were also members of the community (see Table 1).

**Table 1:** Outline of participants

Interviewee ID	Type of participant	Location	Jurisdiction
Community member 1	First Nation Community member	R	
Community member 2	First Nation Community member	R	
Community member 3	First Nation Community member	R	
Community member 4	First Nation Community member	R	
Community member 5	First Nation Community member	R	
Community member 6	First Nation Community member	R	
Community member 7	First Nation Community member	R	
Community member 8	First Nation Community member	R	
Community member 9	First Nations Community member	C	
Community/health worker 1	First Nation Community member/health worker	R	Federal, Saskatchewan on-reserve
Community/health worker 2	First Nation Community member/health worker	R	Federal, Saskatchewan on-reserve
Community/health worker 3	First Nation Community member/health worker	R	Federal, Saskatchewan on-reserve
Physician 1		Saskatchewan	Provincial, Saskatchewan
Physician 2		Saskatchewan	Federal, Saskatchewan on-reserve
Physician 3		Saskatchewan	Provincial, off-reserve, Saskatchewan
TB Program Coordinator		Alberta	Provincial, Alberta
TB Nurse 1	Métis, Neighboring Community member	C	Provincial, off-reserve
TB Nurse 2	First Nation health worker (lives in R as needed)	R	Federal, on-reserve, Saskatchewan
TB Nurse 3	First Nation health worker	R	Federal, on-reserve, Saskatchewan

We used two recruitment strategies to solicit participant interest in our interviews. First, we employed snowball sampling to recruit interviewees from the community. Inclusion criteria were residence in either community R or C, and a minimum age of 18 years at the time of interview. While community R is the focus of this study, recruitment and inclusion criteria was extended to C due to its proximity and members of R often referring to C. Interviews took place in person at the location of the participants’ choosing. Second, we employed purposive sampling to recruit healthcare workers to interview. This was done by directly contacting staff from the TB prevention and Care programs of both Saskatchewan and Alberta. Healthcare workers directly involved with TB care in R and C were approached for interviews including three medical officers of health, four TB workers, and NITHA staff. Of the total of 16 that were approached, 10 participated. Interviews with healthcare workers were performed by both telephones, and in person. In total, we recruited 19 unique interviewees and conducted 22 semi-structured interviews (see Table 1). Three interviewees responded to both types of interviews (community and healthcare worker). Healthcare providers were recruited from two the communities, the Northern Inter-tribal Health Authority in Saskatchewan, and the provincial TB programs in both provinces.

All interviewees were provided an opportunity to review their transcripts for accuracy, ensuring robustness through fidelity. We used NVivo 12 (QSR International) for data coding and analysis. We used thematic analysis, which creates initial codes from the available background information that are revised in an iterative fashion by re-reading transcripts [31]. With respect to questions of mobility, we relied on inductive thematic saturation to organize our data. Through the first few read-throughs of the transcripts, codes were created based on recurring words and themes. We defined thematic saturation as the point when there were no new themes identified from interviews data. In other words, saturation was achieved when motivations and destinations of mobility were repeated [32]. Resulting codes were categorized into overarching emerging themes. Through the exploration of mobility patterns, the major overarching themes were destinations, motivations, seasonal patterns, and lengths of stays. In our complimentary second component, we performed document analysis on current TB guidelines at provincial (Alberta and Saskatchewan), and at national and global levels (see Additional file 1: Appendix 1 for full list). We also applied thematic analysis to these data. In the second component, data were organized by differences and similarities between provinces, current inter-jurisdictional TB policies and the impact of mobility on TB programming. Findings from both analytic components were shared with the Saskatchewan Pathways subcommittee in the community for feedback, and no changes were requested.

## Results

### Overview of TB prevention and care programming in Alberta and Saskatchewan﻿

Although national standards underwrite all provincial TB prevention and care activity in Canada (see the then current 7^th^ Edition of the Canadian TB Standards, 2014), it is up to each of the 13 provinces and 3 territories in Canada to decide how services are delivered. In Saskatchewan and Alberta there were notable similarities and differences in program delivery.

**Fig. 1:**
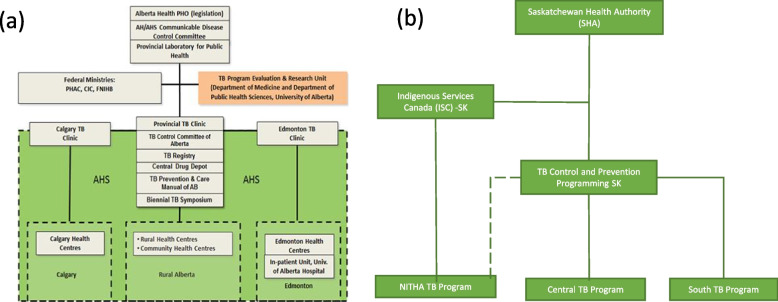
**a** Organization of Alberta TB Prevention and Control Program [33], **b** Organization of Saskatchewan TB Prevention and Control Program

It emerged that TB programming in the two provinces was organized differently. In Alberta, there is one program delivered out of three public health TB clinics, one free standing outpatient clinic in each of the two major cities and a third virtual clinic serving all of rural Alberta, including all reserve communities. They operate within a single province wide health authority (Alberta Health Services). At the time the virtual clinic had a contractual arrangement with the First Nations and Inuit Health Branch of Health Canada to provide TB services on-reserve [33, 34], see Fig. 1a. Saskatchewan also has a single Saskatchewan Health Authority, under which TB Prevention and Care Saskatchewan operates to deliver care province-wide. According to health worker interviews from Saskatchewan (see below), there are three TB program zones, each of which is led by a public health nurse. The *TB Program Worker Handbook* [35] specifies these as the central TB program, the NITHA TB program, and the South TB program (Fig. 1b). NITHA has a contractual arrangement with the First Nations and Inuit Health Branch of Health Canada, Saskatchewan Region, with a mandate to coordinate and provide care for all northern First Nations. According to health worker interviews, NITHA is a third level organization that provides first level support for TB in northern Saskatchewan reserves. These supports include assistance with contact investigations, TB training for TB workers and new community health nurses, and data collection. No comparable organization exists in Alberta. Mobile patients in the region must navigate this jurisdictional quagmire out of necessity.

Until October 26^th^ 2020, “*Saskatchewan used a combination of mobile and telehealth methods*….*there’s no set amount of clinics per year per month or anything, it’s based on needs, on average we were doing them once a month*” [TB Nurse 1, Saskatchewan C]. These mobile clinics travelled to C from the major city of Saskatoon. They served Community C as well as neighbouring communities, including R. Recently these travelling clinics were abandoned in favour of a virtual clinic model similar to that in Alberta [36].

The active treatment procedure is described in the national recommendations, which are standardized and unambiguous and this came across in both the health worker interviews and both Alberta and Saskatchewan provincial TB documents. According to the national standards, once active TB is confirmed the treatment is in two phases, an initial intensive phase which usually lasts two months and a continuation phase which usually lasts four months. Multiple drugs are administered in each phase. According to healthcare workers from both Alberta and Saskatchewan, the duration can range from six months up to 18 months. The length of time of treatment varies according to risk factors, patient compliance, and other life and environmental factors. “*…if they can take their doses all the time and they’re diligent with it takes them 6 months or I've had [a] client go up to 18 months because they're missing so many doses.*” [TB nurse 1, Saskatchewan C]. Directly observed therapy (DOT) is provide to all patients in both provinces. Healthcare workers in Alberta and Saskatchewan described DOT as an opportunity to build trusting relationships with their patients and create a positive experience. TB workers performing DOT supervision act as a point of support while simultaneously promoting treatment adherence. Under exceptional circumstances, medication for the cure of active TB disease in Alberta and Saskatchewan may be self-administered.

In Sect. 60 of *TB Prevention and Control Saskatchewan Clinical Policies and Procedures *[37] home isolation is commonly recommended during the infectious period rather than isolation in a treatment facility. The alternative to home isolation is isolation in a hospital, often at a great distance from the patient’s home. Hospital isolation may be necessary to manage severe or highly infectious forms of disease, complications of the disease, drug intolerance or reactions. Hospitalization for purposes of respiratory isolation was more common in Alberta than Saskatchewan.

In both provinces contact investigations are routinely performed for all pulmonary cases. In addition to the names of individual close, particularly household, contacts, these investigations include gathering information on places of residence, work, and recreation, as well as the timing and duration of exposures. Contacts are grouped into high priority, medium priority, and low priority based on their risk of being infected and progressing to active disease. It is often difficult to determine the exact period of infectiousness of the index case, but generally it is recommended to consider starting contact investigations from three months before the onset of respiratory symptoms. During interviews, the complexities and substantial resources required for contact investigations were emphasized.

*Yes it can be quite huge, which spills over usually from the province (off-reserve community) on to reserve so you get fewer contacts, and you can go up to 100 or 150 people on a contact trace…… they have to all be notified that at some point ... that they've all been in contact of an active case of TB* [TB nurse 2 in R, dual community/health care provider interviewee]

A notable programmatic difference between Alberta and Saskatchewan exists in the provision of preventative therapy for latent TB infection. In Alberta, preventative therapy is offered to all age groups and is the standard of care [38]. In Saskatchewan, however, only children under the age of 15 are prescribed preventative treatment, with individuals over the age of 15 considered eligible on an individual basis [39]. More recently, the Saskatchewan Health Authority in partnership with the Northern Inter-Tribal Health Authority, implemented a community mobilization initiative to expand TB preventative treatment for all age groups in communities R and C.

These typical features of TB programs are aimed at achieving TB elimination. TB elimination depends on employing, in equal measure, a two-pronged approach to management: 1) prevent disease among those infected, and 2) interrupt transmission of the organism among those with active infectious disease [3, 38]. Yet, each previously described step may be challenged by persons moving in and out of reach as a function of their mobility. The impact of mobility on the delivery of TB services, as witnessed by both patients and providers, is underreported.

### Role of mobility in a First Nation community

Interviews with community members and community healthcare workers revealed that mobility in and out of smaller northern communities is a necessity and a norm. Mobility was identified as very common and essential by both Alberta and Saskatchewan healthcare workers.

*…community of R doesn't have a lot of recreational activities …there's limited resources, lack of education, family, and overcrowding* [dual community member/healthcare provider interviewee 2]

*Some people never feel sick enough to stop (i.e mobility and travel) or really change their behavior (i.e mobility and travel out of community) other than that maybe they have a nagging cough they can’t get rid of, so that's what causes them to present not necessarily, like they can’t do their work or play like normal or it may be slowing them down but not as readily, it kind of varies.* [TB Program Coordinator, Alberta]

Community members travel between different jurisdictions from on-reserve to off-reserve and between provinces. Within the overarching theme of motivations for mobility, it is categorized to stick, push, and pull factors [19] as shown in Table 2. According to community interviews, R is geographically isolated, so mobility is required to address local food security, for entertainment, maintaining kinship, medical care, employment opportunities and educational opportunities (Table 2).

*Definitely shopping because it’s so expensive in the north…… their prices are crazy and they often have sales but it’s not the same, it’s not a nice sale from Superstore [A grocery store found in larger communities]. Fort McMurray in the wintertime is 2 hours away or less because the local ... people use that winter road.* [dual community member/healthcare provider interviewee 1]

**Table 2:** Motivations for mobility out of study community. Push factors (reasons for leaving the community), Stick factors (reasons for staying in or coming back to community) and Pull factors (attractants of destinations)

	Subthemes for motivations	Quotes from the community
Stick Factors (reasons for staying in or coming back to community)	Home Family Traditional activities Culture Language	“Right here in C and R both I consider home, I've grown up here all my life, so I consider it all home, this land” [Community member 1] “My home is where I belong with my family and grandkids.” [Community member 9] “…. I'm able to come home and freely speak my language and where I can have access, when I'm home I have access to my traditional food and I have access to my language and my culture, where I can practice my culture” [Community member 6]
Push Factors (Reasons for leaving the community)	Lack of resources Poor access to essentials Cabin Fever Low socioeconomic status Lack of education opportunities Housing availability and conditions Lack of employment opportunities Family Stigma	“for groceries and stuff, prices are so high in town. So travelling is big.” [Community member 3] “essentially everything is out there, there's very little to do here” [Community member 3] “… because food is expensive here, and I will want to travel if I have a bit of money then I will get out of town just to get more stuff, for what I get here I get double out of town.” [Community member 4] “…community of R doesn't have a lot of recreational activities …there's limited resources, lack of education, family and overcrowding” [Community/Health worker 2] “Travel plays a huge huge role in my life, because we are so far up north and we have to travel for medical care, we have to travel for supplies and basically those are the two reasons we travel and just to get supplies and for medical care.” [Community member 6]
Pull factors (attractants to destinations)	Healthcare Employment opportunities Traditional activities Affordable groceries Entertainment Education opportunities Shopping Family and friends	“Well I have doctor’s appointment, when I have doctor’s appointment in PA, Saskatoon, Meadow [Meadow Lake], Battleford [North Battleford]…… Lately I'm going to PA [Prince Albert], every 3 years I go to PA, I go up there for counselling” [Community member 1] “We have to travel for medical care, we have to travel for supplies” [Community member 3] “Every year in July I go there for they have a pilgrimage in July. It's the third week in July. For the last 27 years I've been going there.” [Community member 5]

In addition, land-based activities such as hunting and trapping, and participating in an annual pilgrimage require mobility out of R. *Every year in July I go there for they have a pilgrimage in July. It's the third week in July. For the last 27 years I've been going there.* [Community member (R) 1].

Throughout all the interviews, mobility as a necessity for healthcare access was emphasized. Interviewees identified mental health support, specialist support, hospitals, and dental care as needs for mobility out of the community.

*Well I have doctor’s appointment, when I have doctor’s appointment in PA, Saskatoon, Meadow [Meadow Lake], Battleford [North Battleford]…… Lately I'm going to PA, every 3 years I go to PA, I go up there for counselling* [Community member (R) 1]

**Fig. 2:**
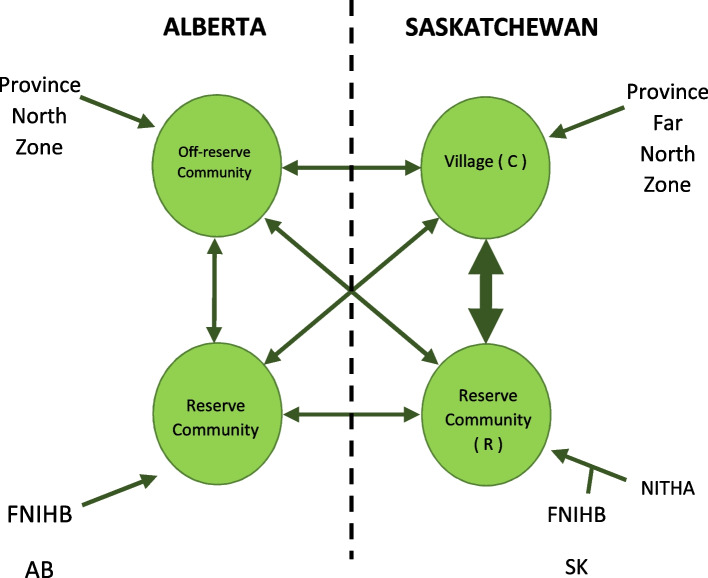
Jurisdictional boundaries and mobility patterns between communities

Common destinations out of R were both inter and intra provincial and both on-reserve (federal), and off-reserve (provincial) (Fig. 2). Moreover, seasonality exerted an influence on mobility of people in the region. For example, mobility out of R to Alberta increased in the winter months with the opening of winter roads that significantly decrease travel time. Interestingly, interviewees from R did not consider C, the neighbouring village, to be a destination but instead a part of their daily life. However, for purposes of TB control, C operates within a separate jurisdiction of care (the province in C, Northern Inter-Tribal Health Authority in R).

### TB care and mobility

Several interviewed community members spoke of the TB experience in the community. The connection between history and contemporary TB was evident in the stories members shared.

*It's interesting you said TB because my aunt years and years ago went to one of those institutions for sanitoriums and she was there for many years, my grandmother and she was finally released but she had told me many horror stories.* [Community member 2]

*….it's a disease that's plagued our communities for so long for like so many years and yet it's kind of like even though we suffer the consequences of TB you know the government doesn't take it as seriously and is not implementing enough measures to make sure that you know that our First Nations communities are safe and are healthy.... And having said that I just feel, when I hear the word TB it's a really threatening subject for me, a real threatening topic.* [Community member 6]

The contemporary TB experience is coloured by history and colonialism. There were similarities between the reasons cited for the high incidence of TB in the community (R) and the reasons cited for mobility, such as access to resources and lack of housing. “*TB is in the community because there's limited resources, lack of education, family and overcrowding, and most important for elimination is education.”* [Community/health worker 3]. Many spoke of needing to travel for healthcare and having appointments in several different communities across SK. *“Travel to me is majority like medical travels usually in Saskatoon”* [Community member 2]. Others spoke of the realities of trying to access medical care from R.

*….I’ve been trying to see a doctor for the last 3 months. Because you’ve got to phone from 9 o’clock in the morning. For the last 3 months I phoned…I want to see the doctor like today not in July or May, I’ve been sick for a while, I don’t want to go up to the hospital (in the city).* [Community member 1]

Traveling for medical care includes urban centers located four to six hours away from community R, including for dental, counselling, hospital, and other medical purposes. Mobility out of the community is a necessity for access to healthcare but may also act as a disruptor of care as identified by health workers.

Mobility across jurisdictions was identified by healthcare workers as a major disruptor of contact investigations – a cornerstone of TB programming. Contact investigations were especially resource intensive and operationally challenging if contacts were identified in multiple locations.

*So if we have somebody [home destination is in R] working in Alberta or just coming back and forth between family…I would gather their information as much information from the index case as I can…..And then I send that off to TB control (Saskatchewan) and then TB control would contact Alberta TB services* [TB Nurse 1 in C]

Once information reaches TB services in Alberta, work gets underway to find and assess all local contacts of the patient with TB. This requires back and forth communication between provinces. The difficulty with performing contact investigations was shared by multiple healthcare workers.

*We try our best, some people do end up falling through cracks still because it's just it can get quite complicated. Especially if someone is taking off to Alberta and then we finally realize a week or two later where they are. And we get phone numbers and so it can get quite complicated and difficult to deal with*. [dual community member/healthcare provider interviewee 2]

The success of contact investigations is a function of the timely completion of each step in the cascade of care. Timeliness might be difficult to achieve across jurisdictions. Interviewees indicated that a lack of direction in this regard in both provincial and national guidelines has resulted in ad-hoc mechanisms for ensuring continuity of care.

The extension of responsibility was especially evident in the provision of DOT, whether it be for active TB or latent infection. Interviews with healthcare workers indicated that DOT care is provided to the patient with flexibility. As such, if a patient on treatment is mobile, healthcare workers attempt to make DOT available to them wherever they are located. Providers of DOT to patients in remote locations are, in this regard, at a great disadvantage. Interviews with healthcare workers not infrequently mentioned driving 50 km or more to provide DOT to patients who had traveled or who reside at a distance. Though there are no standard operating procedures in place, it is common for healthcare workers in the neighbouring communities of R and C to work closely with one another to support patients on DOT. Healthcare workers in C and R extend their jurisdictions to facilitate patients’ needs as well as to provide services to nearby communities.

*I think we just kind of adopted that same mindset that we continue to go out there whenever services, or, you know, like TB meds needs to be given, wherever the person is. Doesn't matter if they're here or in [other nearby communities]* [TB nurse 1, C]

The ad-hoc process identified by Saskatchewan healthcare workers for intra-provincial mobility differs from mechanisms that support mobile patients across provincial borders.

*Even say for instance this guy here that we were supposed to be seeing in (Anonymous community) so every year he goes to work (Anonymous seasonal workplace) in Alberta and he's supposed to be leaving today.….. I will call TB control Saskatchewan and let them know, you know this is the plan they tell us, and we known for about 2 weeks now that he's leaving. Then they will touch base with TB control in Alberta and make arrangements for his med delivery and follow ups need to be done.* [TB nurse 1]

Interviews elucidated the entire process as such: community-based health care workers contact Provincial TB prevention and care in Saskatchewan, who in turn notify Provincial TB prevention and care in Alberta. There is a form that is completed to refer mobile patients between programs. The responsibility for the completion and forwarding of the form is by an individual in the provincial program rather than by healthcare workers directly involved in TB care. However, community-based healthcare workers indicated they were wholly unaware of the existence of these referral forms during interviews, in part perhaps because of turnover of staff. There may also be direct communication between nurses or other front-line workers in the destination community and R. However, these lines of communication were not always clear.

Healthcare workers identified several recommendations for improving communication and coordination between jurisdictions. One physician noted: *“As you know some communities are fly-in only, but most importantly what I think should be happening more effectively is a coordination between these two provinces.”* [Physician 2, Saskatchewan]. Similarly, another physician identified the need for an approach that works beyond the local, and specifically cited the need to consider them within larger systems.

*….the situation when you deal with population health you need to have intersectoral collaboration at different levels…..just want to acknowledge the importance of knowing small geographies….In this kind of whatever geographical system, they need to be more focussed on the community level and at an individual community criteria.* [Physician 3, Saskatchewan]

*Health Canada’s Strategy against TB for First Nations on-Reserve* [40] posits the crucial need to “identify challenges and implement corrective actions as necessary for treatment interruptions and failures, patient mobility and seamlessness of services, disease relapse and drug resistance”. Community participants have identified mobility as a necessity and a challenge for receiving proper healthcare. Currently, much of the responsibility of reaching proper care is the responsibility of community members. Moreover, the aforementioned strategy recommends standardized reporting across jurisdictions, but this has not yet been realized. In 2011 an ad-hoc interprovincial TB Working group (prairies) was established to share information about programmatic challenges, including mobile patients and to collaborate on interventions [41]. Later, this group expanded to become the Canadian TB Elimination Network technical group. It now includes programmatic leaders from all provinces and territories. The existence of these groups and their relationship to delivery of services appeared unknown to healthcare workers interviewed in this study. For example, many of these interviewees called for partnerships and programming through consideration of local epidemiology, though in principle the aforementioned networks fulfill these roles. One significant issue identified by a physician in Saskatchewan is the lack of capacity currently available for creating new policy:

*….the demand for policy and implementation is sort of well in excess of our capacity. So we have to prioritize, and therefore if something is generally working well and it doesn't have a policy, it gets a lower priority because then we're focused on the policies that aren't working well.* [Physician 1, Saskatchewan]

Physicians, nurses, and TB workers continue to call for improved jurisdictional collaboration and policies to address mobility and the disconnection between the multiple jurisdictions in the prairies and nationally. However, to date, this study has not found specific policies and programming to address these calls. Beyond this community members call for an increase in education to tackle TB. *“there's not enough education for our children, there's not enough healthcare present”* [Community member 6].

## Discussion

This study is exploratory in nature. It has identified jurisdictional differences in TB programming, high mobility and poor communication as having the potential to hamper TB elimination efforts. Keeping in mind the current procedures being followed in communities, healthcare workers are taxed to maintain continuity of care despite a lack of supportive guidelines. We have, for example, identified that healthcare workers from northern communities such as R and C extend their jurisdictions informally. In addition, mobility further influences timing and location of transmission. People with infectious TB whose diagnosis was delayed may have extensive networks of contacts across jurisdictions creating more resource intensive and complex contact tracing activities. Contact investigations are an iterative process [3], that require several healthcare professionals and support staff, and many hours of work. The chances of missing contacts of an individual with TB increases with the involvement of multiple jurisdictions especially when coupled with poor lines of communication. Accordingly, reservoirs of infection may become both wider and deeper.

In such contexts of high mobility and jurisdictional morass it is recommended that formal and ongoing communications links be established, and progress monitored. The document analysis and interviews suggest that patient care and TB elimination goals would be served by having programs build on good practices and existing tools and work collaboratively towards removing legal, financial and social barriers to early diagnosis, full treatment and continuity of care across borders. The general principles of cross-border collaboration and continuity of care have been set out by Dara et al. in the context of global migration [42].

The United Nations Declaration on the Rights of Indigenous Peoples (UNDRIP) Article 24.2 states the following, *“Indigenous individuals have an equal right to the enjoyment of the highest attainable standard of physical and mental health. States shall take the necessary steps with a view to achieving progressively the full realization of this right”* [43].

Both our federal and provincial government systems have a responsibility to province health services to reach mental and physical health for Indigenous peoples of Canada. The Truth and Reconciliation Commission of Canada (2015) call to action #20 reminds us of the Federal government’s responsibility to address jurisdictional disputes arising from provision of care for Indigenous peoples in Canada.

As this study illustrates, the lives of Indigenous peoples, both on and off-reserve, are closely connected, and experienced across multiple jurisdictions of care. TB, a primarily respiratory affliction that transmits in the air we share, represents the perfect case study to underscore the confounding nature of complex jurisdictional interactions in health care services [8, 40].

There are several limitations to this study. Firstly, due to ethical concerns and to maintain anonymity of TB patients, the community interviews focused on mobility of the entire population rather than on the mobility of individual patients, specifically. As a result, the findings from this case study provide a general picture of mobility and high-level overview of TB services in the communities of R and C. No community member with active TB chose to participate, though several of those who did participate had family histories of TB and patient perspectives had been sought in an earlier qualitative study that included communities R and C [13–15]. While the community specific details may not be generalizable, the major themes and concepts identified are transferable. Secondly, we relied on snowball sampling, and are likely to have recruited an “information rich” but non-representative cohort of interviewees from the community. Notwithstanding these limitations, this study provides critically important insight into realities of providing TB services, and the needs of border communities that are geographically isolated from major urban centres.

We pose this reflection on the jurisdictional challenges that arise in a federated healthcare system and its fragility in the face of a communicable infectious disease that is historically fraught and weighted with stigma [42]. Strengths of our study include the use of semi-structured interviews to facilitate storytelling, and the use of an iterative and collaborative review during the interpretation of data, methods aimed at grounding the findings in an Indigenous epistemology.

## Conclusion

The lack of a prescribed path of communication within national and provincial guidelines and the current ad-hoc coordination of TB stakeholders/providers points to a need for standardized operation procedures centred on the issue of mobility. The current system functions because of healthcare professionals in Northern communities working beyond their requirements to provide care to an underserved population. To improve current programming, the needs of the healthcare professionals and members in Northern communities need to be heard and considered. Current cross-border programming in World Health Organization European regions and frameworks may provide guidelines on better managing cross-border collaboration. Future research should expand on the findings of this study to explore Indigenous based, community-led research on TB prevention and care programming that improves support and services for Northern communities.

## Supplementary Information

**Additional file 1.**

## Data Availability

The datasets used and/or analysed during the current study are available from the corresponding author on reasonable request.

## Abbreviations

C: Neighbouring northern village
CIHR: Canadian Institutes for Health Research
DOT: Directly-observed therapy
R: Reserve community
TB: Tuberculosis
UNDRIP: United Nations Declaration on the Rights of Indigenous Peoples
