# Supplementary material for: Exploration of inter-jurisdictional TB programming and mobility in a Canadian First Nation community
Source: BMC Public Health. 2022 Dec 14;22:2343. doi: 10.1186/s12889-022-14756-8 (PMC9748898; doi:10.1186/s12889-022-14756-8)
Supplement: Supplementary file 1 — Additional file 1. [file 12889_2022_14756_MOESM1_ESM.docx]

**Appendix 1**

Documents targeted for the review consisted of past and current TB policy statements and guidelines at provincial (Alberta and Saskatchewan), national and global scales. Provincial TB Strategy and Training documents were obtained from the Saskatchewan government website under its Saskatchewan Health Initiatives and from the Alberta website from its Open Government Program. Currently, the Saskatchewan TB program is undergoing major changes, and therefore a complete up-to-date list of guidelines is not available. TB referral forms are not open access and were provided upon requested by TB Control Saskatchewan. National and provincial documents pertaining to Indigenous populations were obtained from the Government of Canada websites and portals.

|  | **Documents Selected** | **Data Analyzed** |
| --- | --- | --- |
| **Saskatchewan** | Saskatchewan Provincial TB Strategy 2013-2018 | - Objectives and goals - Current TB care and prevention programming - Inter-jurisdictional policies and programming |
|  | TB Prevention and Control Saskatchewan Clinical Policies and Procedures | - Current TB care and prevention programming - Inter-jurisdictional policies and programming |
|  | Saskatchewan TB Program Worker Handbook | - Current TB care and prevention programming |
|  | Interprovincial TB Communication Forms (For “follow up/ongoing” communication for short term transient patients for less than four weeks) (Provided by TB Control Saskatchewan) | - Mobility and inter-jurisdictional policies and programming |
|  | Interprovincial TB Referral Form  (For patients who have permanently relocated) (Provided by TB Control Saskatchewan) | - Mobility and inter-jurisdictional policies and programming |
| **Alberta** | Tuberculosis Prevention and Control Guidelines for Alberta | - Objectives and goals - Current TB care and prevention programming - Inter-jurisdictional policies and programming |
|  | Tuberculosis in Alberta Surveillance Report | - Healthcare structure and programming |
| **Policies regarding Indigenous Peoples** | Indian Act | - Indigenous lands and treaty status |
|  | TRC Calls to Action | - Inter-jurisdictional policies |
|  | Health Canada’s Strategy Against TB for First Nations On-Reserve | - Current TB care and prevention - Mobility and inter-jurisdictional policies and programming - Risk factors and SDOH |
| **National** | Canadian Tuberculosis Standards 7^th^ Edition | - Current TB care and prevention programming recommendations - Inter-jurisdictional policies and programming - On-reserve and high incident community recommendations |

**Appendix 2**

**Interview Questions for community participants**

- What does home mean to you?
  - Where do you consider home?
- What role does mobility (or travel) play in your life?
  - Is mobility out of the community important to you?
- How often do you travel out of the community?
  - How long do you travel for?
  - Where do you travel to the most?
- What are the most common routes to Fort McMurray? (Edmonton, Janvier, etc)
  - Do these routes change with the seasons?
- What times of the year do you travel the most?
  - Do you think travel patterns change at different times?
- What are your main reasons for travelling out of the community?
  - (depending on the answers)
    - How important is travel for maintaining **familial** connections? (Garsen Lake)
    - Are travel routes today still reflective of **traditional** hunting routes?
    - Why do you travel out of the community for **work**? How often do you have to travel for work? How often can you come back?
- What does TB mean to you?
  - If you feel comfortable sharing, what experiences have you had with TB?

**Interview guide Healthcare Workers**

- What is the standard procedure for patients presenting with symptoms of TB in (*Location)*?
  - To what extent is the TB standard outlined by your province followed by healthcare workers?
    - Is there a difference between TB programming (care and prevention) in rural compared to urban and city?
  - Where are sputum cultures sent if the patient is from a different jurisdiction?
- If an active TB patient from another province comes to your health location, what is the procedure?
  - If reported, which jurisdiction do you report “mobile” cases to? Is reporting to specific health branches required?
  - What is the inter jurisdictional procedure of reporting active cases for “Status Indian” First Nations?
  - If the patient is still completing their treatment and has travelled between jurisdictions, what are the steps that you take?
  - Can treatment be given in a different jurisdiction?
- Do you utilize DOT? What is the procedure for DOT?
  - What is the procedure for patient isolation? Home isolation?
  - Within the TB strategies, isolation and DOT procedures are different for Saskatchewan and Alberta, how do you think this may impact potentially mobile patients?
- What is the role of the “patient charter of rights” in your community regarding TB treatment?

**Additional questions for Northern Inter-Tribal Health Authority and other policy workers**

- What is Northern Inter-Tribal Health Authority’s role in TB among First Nations?
  - Is the role different for Metis populations?
- What do jurisdictional separation of health procedures mean for TB prevention and care?
